# Investigating the potential therapeutic role of targeting STAT3 for overcoming drug resistance by regulating energy metabolism in chronic myeloid leukemia cells

**DOI:** 10.22038/IJBMS.2022.64138.14121

**Published:** 2022-07

**Authors:** Burcin Tezcanli Kaymaz, Nur Selvi Gunel, Fatma Sogutlu, Neslihan Pinar Ozates Ay, Yusuf Baran, Cumhur Gunduz, Cigir Biray Avci

**Affiliations:** 1 Department of Medical Biology, Ege University Medicine Faculty, 35100, Izmir, Turkey; 2 Department of Molecular Biology and Genetics, Faculty of Science, Izmir Institute of Technology, 35433, Izmir, Turkey

**Keywords:** Chemotherapeutic – resistance, CML, Energy metabolism, Nilotinib, RNAi-based therapeutics, STAT3, Tyrosine kinase inhibitor

## Abstract

**Objective(s):**

STATs are one of the initial targets of emerging anti-cancer agents due to their regulatory roles in survival, apoptosis, drug response, and cellular metabolism in CML. Aberrant STAT3 activity promotes malignancy, and acts as a metabolic switcher in cancer cell metabolism, contributing to resistance to TKI nilotinib. To investigate the possible therapeutic effects of targeting STAT3 to overcome nilotinib resistance by evaluating various cellular responses in both sensitive and nilotinib resistant CML cells and to test the hypothesis that energy metabolism modulation could be a mechanism for re-sensitization to nilotinib in resistant cells.

**Materials and Methods:**

By using RNAi-mediated STAT3 gene silencing, cell viability and proliferation assays, apoptotic analysis, expressional regulations of STAT mRNA transcripts, STAT3 total, pTyr705, pSer727 protein expression levels, and metabolic activity as energy metabolism was determined in CML model K562 cells, *in vitro.*

**Results:**

Targeting STAT3 sensitized both parental and especially nilotinib resistant cells by decreasing leukemic cell survival; inducing leukemic cell apoptosis, and decreasing STAT3 mRNA and protein expression levels. Besides, cell energy phenotype was modulated by switching energy metabolism from aerobic glycolysis to mitochondrial respiration in resistant cells. RNAi-mediated STAT3 silencing accelerated the sensitization of leukemia cells to nilotinib treatment, and STAT3-dependent energy metabolism regulation could be another underlying mechanism for regaining nilotinib response.

**Conclusion:**

Targeting STAT3 is an efficient strategy for improving the development of novel CML therapeutics for regaining nilotinib response, and re-sensitization of resistant cells could be mediated by induced apoptosis and regulation in energy metabolism.

## Introduction

Chronic myeloid leukemia (CML) is a myeloproliferative disorder created by reciprocal translocation of the Abelson (ABL) 1 oncogene on chromosome 9 and the breakpoint cluster region (BCR) on chromosome 22; referred to as t(9;22) thus known as the Philadelphia (Ph) chromosome. This results in the formation of a chimeric fusion protein, p210 kDa BCR-ABL1 oncogene that exhibits aberrant tyrosine kinase activity (1, 2). 

The introduction of tyrosine kinase inhibitors (TKIs) in the treatment of CML is a targeted therapy and is referred to as the gold standard (3). Imatinib, nilotinib, dasatinib, bosutinib, and ponatinib are commercially available TKIs in therapy. Nilotinib was designed to form a better topological fit in the ABL kinase domain of BCR-ABL, referring to an enhanced BCR-ABL inhibition. Although it is thought that treatment failures occur less frequently in the second or third generation of TKIs; a confirmed BCR-ABL mutation described in imatinib-resistant patients also exhibits a higher risk for the development of additional mutations to other TKI treatments, like nilotinib resistance (4, 5). The mechanisms of gained resistance to TKIs involve amplification or mutations in ABL1, up-regulation of P-glycoprotein, down-regulation of drug influx transporters, and finally signaling pathway activation; especially JAK/STAT (6). The activated JAK/STAT pathway stimulates cell proliferation, differentiation, migration, and apoptosis which are critical to cellular processes in hematopoiesis and leukemia development (7). The cascade directly translates an extracellular signal into a post-transcriptional response of downstream targets (8). Among seven STAT family members, especially STAT3 and STAT5 are constitutively activated and transcriptionally overexpressed in various hemato-oncologic malignancies (9). 

Activated STAT3 mediates the expression of various genes in response to cell stimuli, thus playing a key role in cellular processes such as tumor cell growth, proliferation, survival, resistance to chemotherapeutics, and dysregulation of energy metabolism in CML (10). Both pSTAT3 Ser727 and pSTAT3 Tyr705 phosphorylations enhance the transcriptional activity of STAT3 (11–13). pSTAT3 Ser727 supports the maintenance of ETC (electron transport Chain) activity by accumulating STAT3 in the mitochondria and decreases mitochondrial pores’ permeability by stimulating glycolysis, providing cell survival, proliferation, and gain of resistance to apoptosis (14). Besides, STAT3 activity through Tyr705 phosphorylation is related to aerobic glycolysis (15). The metabolic switch from mitochondrial energy metabolism toward glycolysis is one of the main causes of decreased cancer cell susceptibility to chemotherapeutics, thus development of resistance to TKI (16). 

This study aimed to investigate STAT3-mediated nilotinib resistance and the possible mechanisms of re-sensitization to it, by analyzing various cellular responses and metabolic reprogramming in CML cells. We show that nilotinib-resistant CML cells undergo STAT3-dependent survival, apoptosis persistence, transcriptional and translational overexpression, and metabolic alterations; whereas, they could be re-sensitized to nilotinib through siRNA-mediated STAT3 targeting. It has been investigated for the first time that, one of the possible underlying mechanisms for the re-sensitization of resistant cells to nilotinib due to targeting STAT3 might be the regulation of cell energy phenotype of cancer metabolism. 

## Materials and Methods

***Culturing conditions of cells and nilotinib treatment***

Human chronic myelogenous leukemia cell line K562 was purchased from the “German Collection of Microorganism and Cell Cultures” company. They were cultured in RPMI 1640 medium containing 100 u/ml of penicillin-streptomycin, 1% L-glutamine, and 20% heat-inactivated fetal calf serum, at 37 °C in humidified air containing 5% CO_2_. Cells exhibiting 95% survival rates and 85% confluence with the logarithmic growth phase were taken for experimental studies. Nilotinib resistant sub-line was generated from these parental cells in the collaboration’s laboratory, referred to as K562/NiL-50. Briefly, K562 human CML parental cells were exposed to stepwise increasing doses of nilotinib with a starting concentration of 1 nM to a final dose of 50 nM nilotinib; thus, subpopulations of cells were still able to proliferate in the presence of 50 nM nilotinib, which was referred to as resistant K562/NiL-50. These generated nilotinib-resistant cells exhibited higher BCR-ABL expression levels compared with their sensitive parental counterpart. Besides nucleotide sequence analyses of the ABL kinase gene confirmed that these resistant cells did not harbor any mutation in the nilotinib binding region of the gene in resistant cells; thus pointing out, that not mutations, but nilotinib treatment caused the resistance (17). But still, before ongoing with the experiments, a cell proliferation assay was carried out to confirm that the cells were resistant to 50 nM concentration of nilotinib. 

The powder form of nilotinib (Tasigna; Basel, Switzerland) was kindly provided by Novartis Oncology and was dissolved in dimethylsulfoxide (DMSO). 50 nM nilotinib was added to each assay to continue resistance in K562/NiL-50 cells. The anti-STAT3 siRNA transfected and non-targeting negative control parental and resistant cells were used in cell proliferation, apoptosis, real-time RT-qPCR, western blot assays, and metabolic activity analysis. 

***Determining differing expression profiles of STATs in both parental and k562/nil-50 resistant cells***

Differing transcriptional expression levels of STAT3, STAT5A, and STAT5B genes were determined in triplicates by real-time RT-qPCR in both parental and nilotinib resistant cells to determine which one indicated the highest expressional difference among cells. For this purpose, 1×10^6^ cells were collected and total RNA was isolated by following the guidelines of MagnaPure LC RNA Isolation Kit (Roche Applied Science, Mannheim, Germany). The amount and quality of isolated RNAs were measured by a Nanodrop spectrophotometer, and 100 ng RNA was reverse transcribed into cDNA via a Transcriptor High Fidelity cDNA Synthesis Kit (Roche Applied Science, Mannheim, Germany). Relative STAT3, STAT5A, and STAT5B mRNA expression levels were evaluated by the LightCycler Fast Start DNA Master Hybridization Probes Kit (Roche Applied Science, Mannheim, Germany) in LightCycler v.2.0 instrument by G6PDH Housekeeping Gene Set (Roche Applied Science, Mannheim, Germany) manual also referred in our previous study (18). 

The used primer-probe pairs of STAT3 (NM_003150), STAT5A (NM_003152), and STAT5B (NM_012448) are given in Table S1. In brief, the acquired STAT3, STAT5A, or STAT5B target gene’s mRNA copy number was divided to reference gene G6PDH mRNA copy number; obtained from the standard curve that was derived from reference standards ranging between 5×10^2^ and 5×10^6^ copy number of the G6PDH gene as indicated before (18). Evaluating the STAT3 mRNA expression following siRNA treatments was also performed via similar qPCR reactions. 

***siRNA applications for verification of transfection efficiency and targeting STAT3***

Since siRNA transfection optimization was performed in our previous studies, similar siRNA concentrations and transfection reagent amounts were used (19). But still, a transfection efficacy checking experiment was done via transfecting cells with the fluorescent-labeled control siRNA (siGLO Red RISC-Free siRNA, Dharmacon D–001600–01–20) to provide a reliable visual assessment of transfection success under a fluorescent microscope (Olympus, Japan). 

Among investigated STATs, since STAT3 mRNA expression exhibited the highest significant difference in parental and nilotinib-resistant leukemia cells; STAT3 was selected for further analysis. siRNA constructs were purchased from Dharmacon as “On target plus-set of four” specifically designed for targeting STAT3 with four different sequences complementary to STAT3 mRNA. After performing individual success of each sequence, the most efficient siRNA leading the highest suppression rate was selected for modification (20). 

Since siRNAs are difficult to be transfected onto primary cell lines and to reduce off-target effects, chemically synthesized siRNA sequences and transfection reagents were used to target genes of interest for efficient delivery. A chemical modification as “sugar modification” siRNA was conducted for targeting STAT3. The Fluoro*U*ridine substitution with Fluoro*C*ytidine was applied to RNA’s 2′-ribose region which increased the binding affinity of siRNA to STAT3 mRNA with reduced off-target effects; referring to STAT3-FU/FC (Dharmacon, Chicago, IL, USA). siRNA transfection was achieved by following the “HiPerFect Transfection Reagent” (Qiagen, Valencia, CA, and the USA) user manual with 100 nM final concentration siRNA with 2x10^5 ^cells/100 µl in 24 well plates. At the 6^th^ hour of transfection, a full RPMI medium was added to each well for both parental and resistant cells. 

The used anti- STAT3-FU/FC sequences are given below: STAT3-Sense: 2’-F-C.2’-F-C.A.A.2’-F-C.A.A.2’-F-U.2’-FC.2’-F-C.2’-F-.A.A.G.A.A.2’-F-U.G.2’-F-U.2’-F-U.2’-F-U. STAT3-AntiSense: A.2’-F-C.A.2’-F-U.2’-F-U.2’-F-C.2’-F-U.2’-F-U.G.G.G.A.2’-F-U.2’-F-U.G.2’-F-U.2’-F-U.G.G.2’-F-U.2’-F-U. 

Another group of parental and resistant cells was transfected with negative control siRNA (siCONTROL Non-Targeting siRNA 2, D–001210–02–05) with a sequence of 5’- UAAGGCUAUGAAGAGAUAC-3’in order to distinguish sequence-specific silencing from non-specific or non-targeting effects in the RNAi experiment. These control groups of cells were indicated as “non-targeting negative control cells - NC” and to reach optimal silencing effects; both groups of cells were incubated for 72 hr at 37 °C and 5% CO_2_ in the incubator. Non-targeting negative control and anti-STAT3-FU/FC siRNA transfected parental K562 and resistant K562/NiL-50 cells were further used for cell proliferation, apoptosis, real-time RT-qPCR, western blot, and metabolic activity assays. 

***Western blot analysis***

Protein extraction from anti-STAT3 siRNA-transfected and negative control cells was performed according to the “Proteojet Mammalian Cell Lysis Reagent” (Thermo Scientific, Fermentas, USA) instructions. The supplied protein amounts were assessed by the Bradford method by using bovine serum albumin standards ranging between 0.25 and 2 mg/ml concentrations (Thermo Scientific, Fermentas, USA). Finally, 15 μg of each protein extract was resolved at 8 % SDS–PAGE gel and transferred to PVDF membranes using the dry transfer system iBlot (Invitrogen Corporation, Carlsbad, CA, USA). The used primer antibody concentrations were as follows: 1:1000 diluted polyclonal STAT3 (06–596, Upstate), pSTAT3 Tyr705, pSTAT3 Ser727, and β-actin (#9131, #9134, and #4967, Cell Signaling Technology, Beverly, MA, USA, respectively). Primary antibody incubation, blotting, and secondary antibody incubation steps were performed using the western blot Chromogenic Detection Kit (Invitrogen Corporation, Carlsbad, CA, USA). Quantitative detection of proteins was evaluated with a gel view using Image J 1.46r. software.

***Cell proliferation assay XTT in non-targeting negative control and siRNA transfected cells***

Cell viability and proliferation of non-targeting negative control and anti-STAT3-FU/FC siRNA transfected K562 and K562/NiL-50 cells were determined by using the Cell Proliferation Kit II XTT assay (Roche Applied Science, Mannheim, Germany). K562 and K562/NiL-50 cells were either transfected with anti-STAT3-FU/FC siRNA or non-targeting negative control siRNA in the absence or presence of increasing doses of nilotinib ranging between 0.5 - 500 nM for resistant, 0.5 - 250 nM for parental cells, in triplicates. After incubating at 37 °C and 5% CO_2_ for 72 hr, the XTT reagent was added to each well. The absorbance of each sample was measured spectrophotometrically by a microplate reader at 490 nm (Thermo, Vantaa, Finland) at the end of 4 hr of incubation. The obtained data were evaluated with the CalcuSyn v.2 software, cell proliferation curves were generated and IC_50_ values were calculated for both K562 and K562/NiL-50 cells.

***Apoptotic analyses: Annexin V method***

Five hundred thousand of the parental K562 NC siRNA treated control and anti-STAT3 siRNA treated K562 cells and their counterparts resistant K562/NiL-50 cells were incubated for 72 hr and washed 2 times with 1 × PBS. Early and late apoptotic, necrotic, and alive cells were determined according to FITC Annexin V Apoptosis Detection Kit (BD Pharmingen; USA) protocol by BD Accuri™ C6 Flow Cytometer (New York NY, USA). 

***Determining mitochondrial oxygen consumption and glycolysis rates in resistant cells***

The effect of STAT3 inhibition on cellular metabolism upon K562/NiL-50 NC siRNA treated control and STAT3-silenced-K562/NiL-50 cells were determined by the Seahorse XFp cell energy phenotype test according to the protocol supplied by the manufacturer (Agilent Technologies; USA). This test basically determines the energy phenotype of the cells by measuring the OCR in the surrounding cell for “mitochondrial respiration”, and by measuring ECAR for the “glycolysis” status. Seahorse XFp Energy Phenotype Test Kit reveals the metabolic behavior of cells under basal and stressful conditions via using the mitochondrial respiration and glycolysis rates due to measuring two basic parameters of cell energy metabolism such as basic phenotype, stressed phenotype in Agilent Seahorse XF Analyzer instrument (Santa Clara, CA 95051 United States). Seahorse XF instrument measures the oxygen consumption rate (OCR) and extracellular acidification rate (ECAR) of live cells in a multi-well plate to determine key cellular functions such as mitochondrial respiration and glycolysis and the obtained data are analyzed in a Seahorse XFe Analyzer.

***Statistical analysis***

The IC_50_ cytotoxic doses of Nilotinib for both cells were calculated with the CalcuSyn v.2 software. Gene silencing experiments were triplicated and the average of relative expression values was taken at the end of qRT-qPCR analyses as a fold change of relative quantification. For apoptosis, western blot and cell energy metabolism assays, Student’s t-test, and one-way ANOVA analysis were performed. The *P*-value of <0.05 was set as statistically significant by GraphPad Prism v.8.0.1. software. 

## Results

***K562/NiL-50 cells are resistant to 50 nM nilotinib treatment***

**Figure 1:**
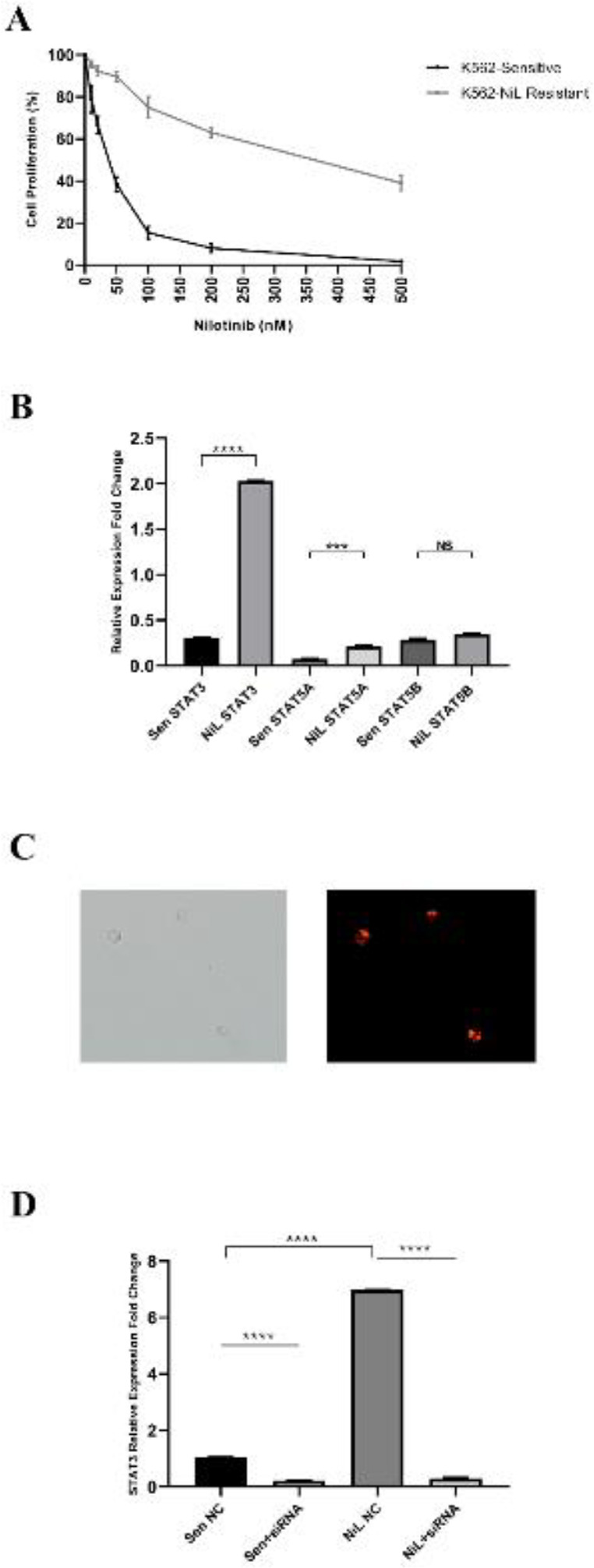
Confirmation and defining target gene studies: 1A) K562/NiL-50 cells are resistant to 50 nM Nilotinib treatment, while 89.3% of the resistant cells were alive under 50 nM nilotinib concentration; it was 38.3% for their parental counterparts. 1B) Differing expression profiles of STATs in both parental and K562/NiL-50 Resistant cells. mRNA expression profiles of STAT3, STAT5A, STAT5B in sensitive (Sen) and nilotinib resistant (NiL) K562 cells revealed by RT-qPCR and STAT3 expression significantly exhibited the highest with 6.6 fold increase; whereas STAT5A with 2.84 fold increase in resistant cells compared with their sensitive counterparts; but STAT5B expression did not differ among cells. ****P<0.0001, *** *P*<0.001, NS: Not significant. 1C) Confirming siRNA transfection efficiency. The cellular siRNA uptake images were taken for a duration of the 24th of siGLO Red RISC-Free siRNA treatment of resistant cells by fluorescein microscopy and phase-contrast + fluorescein microscopy images merged × 40 magnification. The red fluorescent signal localized to the nuclei indicates a perfect signal of efficient siRNA uptake. 1D) STAT3 mRNA expression is efficiently suppressed due to siRNA Transfection. STAT3 mRNA expression in NC siRNA treated and anti-STAT3 siRNA treated sensitive cells (Sen NC vs Sen+siRNA) and their resistant counterparts (NiL NC and NiL+siRNA) were detected. STAT3 mRNA relative expression was significantly suppressed by -4.83-fold in sensitive cells and -23.3-fold in resistant cells compared with their siRNA transfected counterparts; the suppression fold change was 6.87 higher in resistant cells. ****P<0.0001 for all by using one-way Anova

A cell proliferation XTT assay was carried out to confirm that the resistant cells referred to as K562/NiL-50 were resistant to 50 nM of nilotinib treatment. It was established that 89.3% of the K562/NiL-50 cells were alive under 50 nM nilotinib exposure; whereas 38.3% of the sensitive cells survived at the same nilotinib concentrations (Figure 1A). K562/NiL-50 cells were referred to as resistant to nilotinib treatment. 

***Differing transcriptional expression profiles of stats: STAT3 is significantly up-regulated in K562/NiL-50 resistant cells***

The analyzed STAT3, STAT5A, and STAT5B expression profiles indicated that while STAT5B expression did not differ (*P*>0.05); STAT5A was 2.84-fold up-regulated (*P*<0.001), and 6.6-fold increase (*P*<0.0001) was detected for STAT3 in K562/NiL-50 resistant cells compared with parental ones (Figure 1B). Since STAT3 exhibited the most differing expression profile in resistant cells; STAT3 was selected as the main target for further analysis.

***Confirmation of transfection efficiency***

siRNA transfection efficiency was detected via fluorescently labeled siRNA and visualized that all of the cells were transfected with siRNA based on the red spots seen in the nucleus of the cells (Figure 1C). The ongoing silencing experiments were performed for targeting STAT3.

***Effects of RNAi-mediated STAT3 silencing in parental and resistant cells: STAT3 mRNA expression is efficiently suppressed***

The suppression rates of STAT3 mRNA levels due to siRNA transfection were determined by real-time qRT-qPCR in both parental and resistant cells. While the STAT3 gene expression level was decreased to 20.7% (4.83-fold; *P*<0.0001) in sensitive parental cells, the suppression rate was 95.1% (23.3-fold; *P*<0.0001) in nilotinib-resistant cells when compared with their siRNA transfected counterparts. When expression difference was compared within parental and resistant cells; STAT3 was 6.87-fold more down-regulated (*P*<0.0001) in resistant cells (Figure 1D). 

***Total STAT3, pSTAT3 Tyr705, and pSTAT3 Ser727 protein expression levels were also repressed due to siRNA treatment***

**Figure 2:**
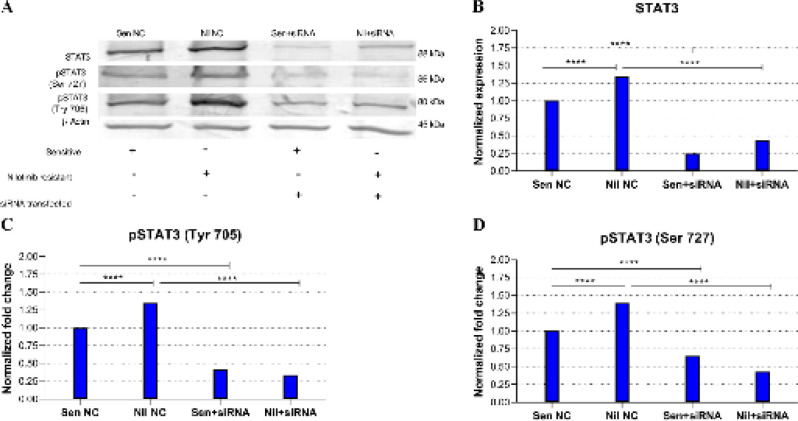
Western blot analysis results of total STAT3 and Tyr705 or Ser727 phosphorylated STAT3 levels in parental and resistant cells: A) Protein gel image of total STAT3, pSTAT3 Tyr705, and pSTAT3 Ser727: expressional increases were detected in resistant cells compared with sensitives and expressional down-regulations were observed due to siRNA applications. B) Graphical presentation of total STAT3 Expression: Following anti-STAT3 siRNA treatment, sensitive and resistant cells’ total STAT3 protein expression levels were significantly reduced by 4.0-fold and 3.04-fold; respectively. C) Graphical presentation of pSTAT3 Tyr705 Expression: following anti-STAT3 siRNA treatment, sensitive and resistant cells’ pSTAT3 Tyr705 expression levels were significantly reduced by 2.38-fold and 3.97-fold, respectively. D) Graphical presentation of pSTAT3 Ser727 Expression: following anti-STAT3 siRNA treatment, sensitive and resistant cells’ pSTAT3Ser727 expression levels were significantly down-regulated by 1.54-fold and 3.23-fold, respectively

The expressional changes of total STAT3, pSTAT3 Tyr705, and pSTAT3 Ser727 protein levels for both parental and resistant leukemic cells were determined (Figure 2A). 

*Comparison of NC siRNA treated resistant and parental sensitive cells’ expression profiles*

As normalized to housekeeping beta-actin expression, while each NC siRNA transfected control groups’ total STAT3, pSTAT3 Tyr705, and pSTAT3 Ser727 protein expression levels were taken as 1.0 in parental sensitive cells; the levels were detected to be 1.34, 1.35, and 1.39, respectively (*P*<0.0001 for both,) in resistant NC siRNA treated cells. So, it is confirmed that each analyzed STAT3 protein expression level was higher in resistant cells compared with parental ones as a correlation of transcriptional expression profiles (Second compared with first columns in Figure 2B; Figure 2C; Figure 2D). 

*Comparison of anti-STAT3 siRNA treated parental sensitive cells expression profiles*

STAT3 protein expression levels were analyzed following anti-STAT3 siRNA treatment exposed to parental sensitive cells. As for the results, since Sen NC expression profiles were taken as 1.0 for both three STAT proteins, the total STAT3 expression level was reduced to 0.25 (4.0-fold), pSTAT3 Tyr705 was reduced to 0.42 (2.38-fold) and pSTAT3 Ser727 to 0.65 (1.54-fold) in anti-STAT3 siRNA transfected parental cells indicated as Sen NC and Sen+siRNA (Third compared with first columns in Figure 2B; Figure 2C; Figure 2D) (*P*<0.0001 for all). 

*Comparison of anti-STAT3 siRNA treated resistant cell expression profiles*

Since NiL NC total STAT3 expression was detected as 1.34; it was reduced to 0.44 (3.04-fold) due to anti-STAT3 siRNA treatment. As NiL NC pSTAT3 Tyr705 was 1.35, it was reduced to 0.34 (3.97-fold) and NiL NC pSTAT3 Ser727 was detected as 1.39, it was reduced to 0.43 (3.23-fold) in anti-STAT3 siRNA transfected resistant cells indicated as NiL NC and NiL+siRNA. (Forth compared with second columns in Figure 2B; Figure 2C; Figure 2D) (*P*<0.0001 for all).

Due to siRNA treatments, all analyzed STAT3 protein expression levels were decreased at the translational level both in parental and resistant cells. The highest suppression rate detected in resistant cells was the pSTAT3 Tyr705 expression with a 3.97-fold decrease.

***Nilotinib chemosensitivity is increased in parental and nilotinib resistant cells following siRNA treatment***

**Figure 3:**
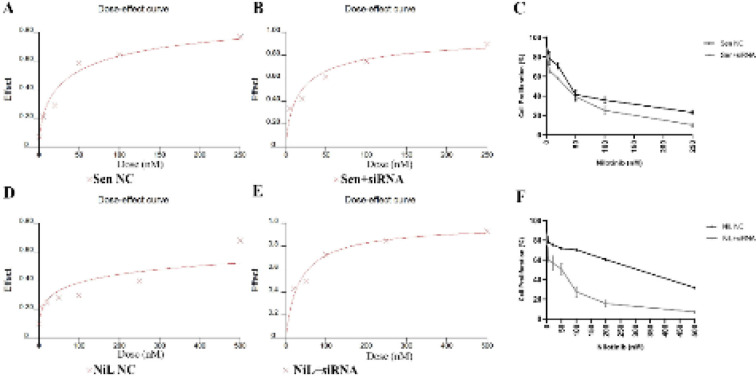
Dose-effect analyses and cell proliferation curve graphs showing IC_50_ values for Nilotinib: Dose-effect curve was generated by CalcuSyn 2.1 software for cells treated by various dilution ratios of Nilotinib doses. A) NC siRNA treated sensitive (Sen NC) cells IC_50 _dose was calculated as 40.53 nM. B) anti-STAT3 siRNA transfected sensitive cells (Sen +siRNA) as 19.85 nM. C) Effect of dose increasing treatments on cell proliferation in Sen NC and Sen+siRNA groups. D) NC siRNA treated resistant cells (NiL NC) as 384.66 nM and E) anti-STAT3 siRNA treated resistant cells (NiL+siRNA) as 35.51 nM. F) The effect of increasing dose treatments on cell proliferation in NiL NC and NiL+siRNA groups

The indicated nilotinib IC_50_ values were 40.53 nM (r: 0.9853) for parental K562 (Figure 3A) and 384.66 nM (r: 0.9146) for resistant K562/NiL-50 cells, respectively (Figure 3A; Figure 3B; Figure 3C; Figure 3D). The assay was also performed following siRNA treatments to define whether silencing STAT3 sensitized especially resistant cells under lower nilotinib concentrations. In anti-STAT3-FU/FC siRNA transfected cells while IC_50_ value was decreased to 19.85 nM (r: 0.9636) in parental cells (Figure 3B; Figure 3C), it was decreased to 35.51 nM (r: 0.9817) in resistant cells (Figure 3E; Figure 3F). So, sensitivity was significantly increased in both K562 and K562/NiL-50 cells. The reduction in IC_50_ values indicated that, while parental cells became 2.04-fold more sensitive to nilotinib, the resistant cells exhibited 10.83-fold increased sensitivity and drug response towards nilotinib due to STAT3 silencing. 

***Apoptosis is dramatically induced both in parental and resistant cells due to silencing STAT3***

**Figure 4:**
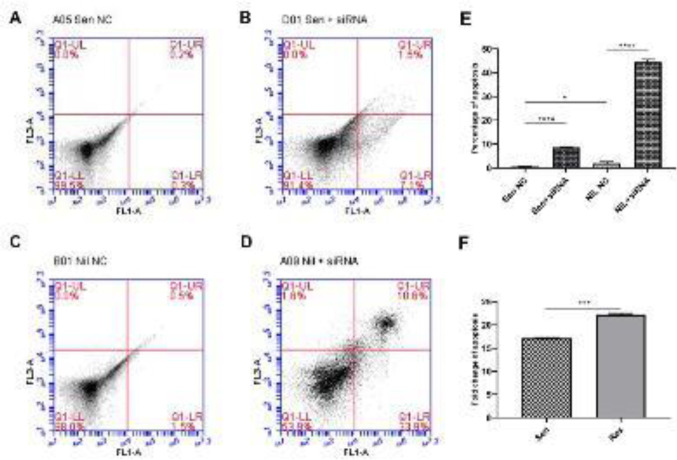
Apoptosis assay results of flow cytometer analysis. A) NC siRNA treated sensitive (Sen NC), 0.5% apoptosis. B) Anti-STAT3 siRNA treated sensitive (Sen +siRNA), 8.6% apoptosis. C) NC siRNA treated resistant (NiL NC), 2.0% apoptosis. D) anti-STAT3 siRNA treated resistant (NiL+siRNA); 44.5% apoptosis. While the sensitive cells were 17.2-fold, the resistant cells exhibited a 22.25-fold increase in apoptosis due to targeting STAT3

Silencing effects of STAT3 upon apoptosis in parental and nilotinib resistant cells were determined. A total of early and late apoptosis rates were detected as follows: 0.5% in NC siRNA treated parental sensitive and 8.6% in its STAT3 silenced counterpart, with a priority of early apoptotic cells with 7.1% (Figures 4A, 4B). As for nilotinib-resistant cells, apoptosis was determined as 2.0% in NC siRNA treated cells [1.5% early, 0.5% late apoptotic); whereas it is increased to 44.5% [33.9% early, 10.6% late apoptotic] due to STAT3 silencing (Figures 4C, 4D). Overall, these results indicate that, suppression of STAT3 induced apoptosis at high levels with 17.2-fold in the parental and 22.25-fold in nilotinib-resistant CML cells (Figures 4E, 4F)

***Targeting STAT3 in K562/NIL-50 resistant cells modulated cell energy phenotype and switched the metabolic phenotype toward oxidative phosphorylation***

**Figure 5:**
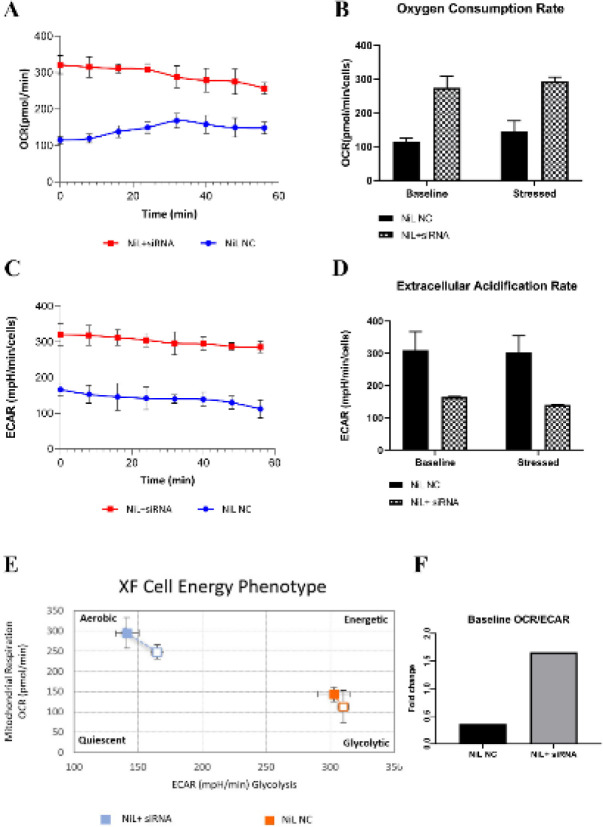
XF cell energy phenotype assay results. A, B) Alteration in OCR (oxygen consumption rate gives cells mitochondrial oxygen rate) OCR was found as 114.23 pmol/min/cells in NC siRNA treated cells and 247.44 pmol/min/cells in its STAT3 silenced counterparts. Following stressor application: OCR was 145.03 pmol/min/cells in NC siRNA treated cells, and 293.97 pmol/min/cells in STAT3 silenced cells. C, D) ECAR (extracellular acidification rate- gives cells glycolysis rate) values before (ECAR was detected as 309.66 mpH/min/cells in NC siRNA treated cells and 165 mpH/min/cells in anti-STAT3 siRNA applied cells) and after stressor application (FCCP\Olygomycin) (after stressor application, ECAR was decreased to 302.33 mpH/min/cells in NC siRNA treated cells and 141 mpH/min/cells in anti-STAT3 siRNA treated cells). E) Orange and blue hollow square: OCR and ECAR values at baseline, orange, and blue-shaded square: energy tendency after stressor was applied. F) Rating of baseline OCR to baseline ECAR in NiL NC and NiL+siRNA groups

While the basal metabolism of OCR was detected as 114.23 pmol/min/cells in NC siRNA treated cells, it was 247.44 pmol/min/cells in its STAT3 silenced counterparts. Also, the mitochondrial respiration referring to the oxidative phosphorylation was increased to 145.03 pmol/min/cells in NC siRNA treated cells, and to 293.97 pmol/min/cells in STAT3 silenced cells following stressor application (Figure 5A; Figure 5B). When glycolytic metabolism potential was analyzed, ECAR was found to be 309.66 mpH/min/cells in NC siRNA treated cells and 165 mpH/min/cells in anti-STAT3 siRNA applied cells, whereas following stressor application, they were decreased to 302.33 mpH/min/cells in NC siRNA treated cells and 141 mpH/min/cells in anti-STAT3 siRNA treated cells, respectively (Figure 5C; Figure 5D). When the detected OCR values in basal metabolism were divided into ECAR values, the fold change was 0.36 in NC siRNA treated cells; whereas it was determined as 1.66 in siRNA treated cells (Figure 5E; Figure 5F). This obtained data pointed out that, suppression of STAT3 in K562/NIL-50 resistant cells modulated cell energy phenotype and switched the glycolytic metabolism towards oxidative phosphorylation; thus, silencing STAT3 mediated metabolic reprogramming from resistance to sensitivity.

**Table 1:** Nucleotide sequence of the primer-probe pairs used for the STAT3, STAT5A, and STAT5B genes in RT-qPCR analysis

STAT3	Forward primer	**5'- ACCAACAATCCCAAGAATGT-3'**
	**Reverse primer**	5'-CGATGCTCAGTCCTCGC -3'
	**Fluorescein**	5'- TCAAGTGGCCGAGGTCCTGA-FL-3'
	**LC Red 640**	5'-CTGGCAGTTCTCCTCCACCACCA –p-3'
STAT5A	**Forward primer**	5'-GAAGCTGAACGTGCACATGAATC- 3'
	**Reverse primer**	5'-GTAGGGACAGAGTCTTCACCTGG -3'
	**Fluorescein**	5'-ACAGGACTGTGAACTTCTCCTCTGTCACGG-FL-3'
	**LC Red 640**	5’-CTCTGCACCCCGCCGGTCAG –p-3'
STAT5B	**Forward primer**	5'- AGTTTGATTCTCAGGAAAGAATGT -3'
	**Reverse primer**	5'- TCCATCAACAGCTTTAGCAGT-3'
	**Fluorescein**	5'-TTGGGAGACTTGAATTACCTTATCTACGT –FL-3'
	**LC Red 640**	5’-TTCCTGATCGGCCAAAAGATGAA-p-3'

In each analysis total STAT3, Tyr705, and Ser727 phosphorylated STAT3 protein expression levels normalized according to the intensity of beta-actin bands. Both STAT3 and pSTAT3 protein expressions were down-regulated due to STAT3 gene silencing in sensitive and resistant cells, respectively. ****P<0.0001

for all and were detected to be significant according to one-way Anova

Apoptotic ratio and fold change among cells: E) Presents the apoptotic percentage of all groups and F) Comparison apoptosis fold change within Sen NC siRNA vs Sen+siRNA and NiL NC siRNA vs NiL+siRNA cells. Apoptosis was 1.29-fold higher induced in resistant cells. A double asterisk indicates P<0.05 within these indicated groups. [Four quadrants; late apoptosis in the upper right corner, necrosis in the upper left corner, early apoptosis in the lower right corner, and percentage illustration of viable cells in the lower-left corner]

## Discussion

Gained chemotherapeutic drug resistance remains one of the most important restricting obstacles in the cure of hematological malignancies, especially towards TKIs in CML. Thus, researchers focus on identifying new and therapeutic targets in the molecular basis of CML, like STATs (21). This study defines a STAT3 overexpression mediated surveillance, apoptotic, transcriptional, translational, and metabolic regulation in TKI nilotinib sensitive and resistant K562 cells, *in vitro.* Clarifying the underlying mechanism of STAT3-mediated nilotinib resistance provides new potential therapeutic targets to eliminate these TKI-resistant cells by reducing leukemic cell survival, enhancing leukemic cell apoptosis, and modulating cell energy phenotype, which in turn increases the chance of re-sensitization to nilotinib. Consequently, we researched for understanding the mechanism of STAT3-mediated nilotinib resistance and focused on the differing energy metabolism modulation of resistant cells; which might be potentially a pivotal reason for the gain of re-sensitization to nilotinib due to targeting STAT3. 

We observed that both parental and resistant cells –but priorly resistant ones– exhibited higher IC_50_ doses of nilotinib response, increased leukemic cell survey, significantly up-regulated STAT3 mRNA transcript, and total pSTAT3 Ser727 and Tyr705 protein expression levels; whereas, targeting STAT3 via siRNA applications induced anti-cancerous properties of the cells: both parental and resistant cells; especially the resistant cells displayed significant and encouraging response to nilotinib treatment by exhibiting more than ten times chemosensitivity and dramatically down-regulated STAT3 mRNA and total and pSTAT3 Tyr705 and Ser727 protein expression levels. Similarly, Ma LD *et al*. have reported that silencing STAT3 sensitized K562 leukemic cells, inhibited leukemic cell proliferation, and induced apoptosis (22). Bewry* et al*. have also used RNAi technology to silence STAT3 in imatinib-resistant cells and reported that reducing STAT3 expression levels had sensitized K562 cells to imatinib. This resistance was correlated with this increased STAT3 mRNA, total and pSTAT3 Tyr705 and Ser727 expression levels (23). Many other studies also suggested that STAT3 overexpression promoted leukemic cell proliferation and that the inhibition of STAT3 activity could reduce leukemic cell survival and proliferation (7, 9, 10, 24–27). 

The cytotoxic efficacy of chemotherapeutics is evaluated according to their ability to activate the apoptotic pathway, but dysregulations and uncontrolled leukemic cell proliferation cause a loss of sensitivity to TKI (28). Besides, STAT3 phosphorylation either directly regulates apoptosis by increasing the expression of anti-apoptotic genes and apoptosis inhibitors or indirectly affects cell survival by changing energy metabolism. Constitutively active pSTAT3 Tyr705 decreased oxidative phosphorylation and changed metabolic phenotype to aerobic glycolysis and led to the leukemic cells’ resistance to apoptosis (29). The surviving leukemia cancer cells that were dependent on oxidative phosphorylation to efficiently produce energy rely on STAT3 were localized to mitochondria, and when pSTAT3 Ser727 was localized to the inner membrane of mitochondria, reactive oxygen species were released, detoxified the cell, and by reducing caspase 3 activation, leukemic cells were protected from apoptosis (30–32). 

Since apoptotic, STAT3 protein expressional and metabolic regulations seemed to be dependent on their interactions within each other, and CML cells relied on glycolytic and mitochondrial functions to provide high energy and metabolic needs for escaping apoptosis, we initially analyzed whether both nilotinib sensitive and resistant leukemic cells showed resistance to apoptotic cell death and then, we checked the correlation between low apoptosis rates and high phosphorylated forms of STAT3 protein expression levels (33–35). Secondarily, the cellular responses of the cells were evaluated due to silencing STAT3 and finally, the metabolic reprogramming was evaluated in resistant cells. The detected apoptotic leukemic cell rate was very low at 0.5% in sensitive cells and 2.0% in its resistant counterparts, and pSTAT3 Tyr705 and Ser727 expressions were up-regulated in our resistant cells. But when STAT3 was suppressed, leukemic cell apoptosis was highly and significantly induced both in sensitive and resistant cells with 17.2-fold and 22.25-fold increases, respectively, and protein expression levels were down-regulated, highest for Tyr705, then Ser727 in resistant cells. This data showed similar outcomes as given in the literature above; persistence apoptosis was in line with up-regulated STAT3 protein expression levels.

As for cancer metabolism, cancerous cells have evolved their metabolic behavior towards 50% of glycolysis; even in the presence of oxygen, they still prefer the glycolytic pathway (36, 37). Investigations indicate a correlation between STAT3 signaling and cancer cell metabolism. These studies support that up-regulated STAT3 activity gave rise to the expressional triggering of the genes that promote the cancer phenotype with advanced metabolic changes (11, 38–40). The cell energy phenotype change in tumor cells caused cancer progression and resistance to chemotherapeutics; which were specifically related to up-regulated phosphorylated STAT3 protein expression levels (21, 41, 42). Since our nilotinib-resistant cells also displayed increased protein expression levels of pSTAT3 residues, referring them to be in a glycolytic state; we checked whether metabolic reprogramming will be occurred due to silencing STAT3. We determined that oxygen consumption rate was increased giving rise to mitochondrial respiration; and, even in the presence of the stressor, maximal oxidative phosphorylation capacity was induced. When glycolysis efficiency was evaluated, we determined that the extracellular acidification ratio was much higher in NC siRNA treated resistant cells compared with their STAT3 silenced counterparts and the maximal glycolysis capacity was non-significantly decreased according to basal metabolism in both groups. These results indicated that nilotinib-resistant K562 cells initially preferred aerobic glycolysis instead of mitochondrial oxidative phosphorylation; but due to targeting STAT3, aerobic glycolysis was reduced in correlation with decreased protein expression levels in resistant cells. One possible mechanism of this metabolic switching might be due to the balanced ETC activity that resulted in increased mitochondrial respiration after down-regulation of pSTAT3 Tyr705 and pSTAT3 Ser727 expressions in nilotinib-resistant cells. It has been similarly reported that constitutively active STAT3 promoted an aerobic glycolysis-like state; especially pSTAT3 Tyr705 residue played a role in switching energy metabolism to aerobic glycolysis (40, 43, 44). In their study, Demaria* et al*. also pointed out that the decrease in protein expression of total and pSTAT3 Tyr705 levels reduced aerobic glycolysis independent of mitochondrial activity. In a current study, Patel* et al*. showed that STAT3 in CML stem and progenitor cells regulated the development of resistance to tyrosine kinase inhibitors by metabolic programming, and metabolism in TKI-persistent leukemic cells was reported to be in a glycolytic state unless inhibition of STAT3 using a small molecule inhibitor. Besides CML, Zhang* et al*. reported that expressional down-regulation of STAT3 by targeting led to the metabolic phenotype of the cells from aerobic glycolysis to mitochondrial respiration in head and neck squamous cell carcinoma (44, 45). 

All obtained data showed for the first time that due to STAT3 overexpression, nilotinib-resistant leukemic cells metabolism referred to a glycolytic state; whereas targeting STAT3 by siRNA treatment therapeutically re-sensitized them by switching energy metabolism from aerobic glycolysis to mitochondrial respiration in K562 cells. Our findings should be an initial step to guide future studies by showing the interaction between STAT3 and cancer cell energy phenotype of the glycolytic pathway; which might be the underlying mechanism gain of resistance to TKI nilotinib.

## Conclusion

Targeting STAT3 via siRNA applications seems to be a potent strategy for improving novel CML therapeutics as overcoming nilotinib TKI resistance, and re-sensitization of nilotinib in resistant cells could be mediated by inducing apoptosis and modulating cell energy phenotype.

## Authors’ Contributions

BTK Provided the idea and design; NSG and FS were responsible for acquisition of data; CG and CBA Provided analysis; BTK, YB, and NPO Interpreted the findings; BTK and FS Helped with writing; all authors edited and confirmed the final form. 

## Financial Disclosure Statement

There are no financial supports of interest to disclose. 

## Conflicts of Interest

The authors have no conflicts of interest to declare.
